# Histoplasmosis Associated with a Bamboo Bonfire — Arkansas, October 2011

**Published:** 2014-02-28

**Authors:** Dirk T. Haselow, Haytham Safi, David Holcomb, Nathaniel Smith, Kendall D. Wagner, Branson B. Bolden, Nada S. Harik

**Affiliations:** 1 Arkansas Department of Health; 2 Department of Pediatrics, University of Arkansas

On October 27, 2011, the Arkansas Department of Health (ADH) was notified by a northeast Arkansas primary care provider of a cluster of three histoplasmosis cases. On November 4, ADH was notified by a pediatric infectious diseases specialist regarding seven potential cases of pulmonary histoplasmosis associated with a family gathering that included a bonfire that burned bamboo from a grove that had been a red-winged blackbird roost. These reports prompted an outbreak investigation to ensure that the persons involved received appropriate medical care, to identify whether any novel exposures were associated with illness, and to determine whether any factors were associated with hospitalization. The investigation found that, among the 19 attendees at the family gathering, seven were confirmed with histoplasmosis, 11 were probable, and one did not have histoplasmosis.

## Index Cases

Investigators found that two siblings, a boy aged 8 years and a girl aged 5 years had become ill on October 16, reporting vague abdominal pain and a dry cough. One day later, both children developed fever and nonbloody emesis, prompting their parents to seek care for them. The children were determined to be rapid streptococcal antigen–positive and were prescribed amoxicillin for 10 days. During the next 6 days, their coughs worsened and became productive of white sputum. Both continued to be febrile with temperatures ≥104°F (≥40.0°C).

On October 22, the children returned to their primary care provider. Each had a chest radiograph (CXR) demonstrating bilateral diffuse infiltrative disease. Both were diagnosed with pneumonia, admitted to a local hospital, and placed on intravenous azithromycin and ceftriaxone. On October 24, both children were transferred to Arkansas Children’s Hospital for further care.

At the hospital, the two children had increasing oxygen requirements and sustained high fevers. Repeat CXRs demonstrated micronodular density patterns bilaterally and mediastinal lymphadenopathy. Additional history revealed that the children had attended a family gathering 8 days before symptom onset. Participants cut bamboo, made a fort, and burned wood in a small grove that had served as a red-winged blackbird roost. Other attendees were reported to be ill with similar symptoms.

Serum specimens for *Histoplasma capsulatum* yeast and mycelial antibody tests were obtained, and the children were empirically started on itraconazole 5 mg/kg/dose twice daily. Both children improved dramatically with antifungal therapy and defervesced within 48 hours. Both had positive *Histoplasma* yeast and mycelial antibodies and positive serum antigen results. The siblings completed a 3-month course of itraconazole for acute diffuse pulmonary histoplasmosis. Repeat CXRs demonstrated resolution of acute lung findings.

## Epidemiologic Investigation

A retrospective cohort study was performed to determine the extent of the outbreak and risk factors for illness. Cases were sought by asking attendees at the gathering to recall the names of all other attendees. All attendees were interviewed with a standard questionnaire and were offered free serologic testing for *Histoplasma*.

The local county health officer contacted all local primary care providers to assist in case finding. Because histoplasmosis is reportable in Arkansas, case identification also was attempted by reviewing all *Histoplasma*-related laboratory results reported to the ADH communicable disease surveillance system. All persons with suspected histoplasmosis identified in this manner in October and November were contacted to determine whether their illness was related to this outbreak. Clinical records were obtained for all persons who sought care.

The Council of State and Territorial Epidemiologists has not published standard case definitions for sporadic or outbreak-related acute respiratory histoplasmosis. In Arkansas, cases are considered to be confirmed if the patient has a measured fever ≥101°F (≥38.3°C), and either cough, chest pain, shortness of breath, or abnormal CXR, and at least one positive culture, antigen, or serologic test for *Histoplasma*. Cases are considered probable if the patient has symptoms consistent with histoplasmosis (self-reported fever and either cough, chest pain, or shortness of breath) and at least one positive culture, antigen, or serologic test for *Histoplasma*.

Because subclinical illness and illness for which no histoplasmosis tests were performed were observed in this outbreak, when a confirmed case was identified, the definition of a probable case was broadened to include any person exposed to the site or event who also had clinical features of fever ≥101°F (≥38.3°C) and at least one of cough, chest pain, shortness of breath, or abnormal CXR within 3 weeks of exposure, even in the absence of laboratory testing for *Histoplasma*.

All attendees were asked to provide blood for analysis. If provided, serum was sent to ARUP Laboratories, a national reference laboratory in Salt Lake City, Utah, for assessment of quantitative titers for *Histoplasma* yeast and *Histoplasma* mycelial immunoglobulin G.

Investigators learned that 19 persons, aged 4–62 years, had attended the family gathering during October 7–8, 2011. Of those persons, 12 were male. The majority of attendees were children; eight were aged <10 years, and five were aged 10–19 years.

The setting was the backyard of a home in a residential area of a small town in northeastern Arkansas, with no construction or excavation projects nearby. The site was approximately one quarter acre in area, of which roughly 25% was wooded and 75% was covered by well-groomed zoysia grass. The wooded area included a tall canopy of white pine and sparse, bamboo grove understory contained within an area measuring approximately 15 feet by 15 feet (4.6 meters by 4.6 meters). The bamboo grove was described as a prominent red-winged blackbird (*Agelaius phoeniceus*) roost. Bat and bird droppings are a common source of histoplasmosis contamination (1). Local residents stated that during annual migrations there were so many birds as to “darken the sky.” No bats were reported.

Activities during the gathering on October 7 included clearing a small patch of bamboo and building a bamboo fort. On October 8, the family built a bamboo bonfire and used it to roast hot dogs. Leaf litter or ash was then raked and children were noted to be playing in the dirt.

The host family had moved to this location 1 month before the gathering. The family had not previously spent extended time in the backyard. Family members had never before cut or burned bamboo. No other attendees had direct exposure to this site previously. One adult was visiting Arkansas for the weekend from an area where histoplasmosis is not endemic.

Attendees were healthy; none reported an underlying pulmonary or immune-related disorder. Four attendees cited one underlying medical condition each: allergic rhinitis (two), attention deficit hyperactivity disorder, and insomnia.

Among attendees, all 19 participated in the bamboo bonfire and cookout, three cut bamboo, three built a small bamboo fort, one raked leaf litter, and eight raked or disturbed bamboo ash. All 19 attendees reported illness after the gathering; however, one attendee was excluded from the case definition because this person reported only a headache and cough. Among the 18 attendees who met the case definition, 16 could recall the date of their illness onset (Figure). Among the 18, the most common signs and symptoms included fever (83%), cough (67%), and shortness of breath (61%) (Table).

## Test Results

CXR results were abnormal for 11 (79%) of the 14 attendees who had CXR performed. Among those 11 persons, *Histoplasma* yeast antibody results were positive for 10, and mycelial antibody results were positive for eight. For three of six attendees tested, serum antigen test results were positive, and urine antigen test results were positive for two of three attendees who had that test performed. No attendees had bone marrow biopsies, tissue biopsies, or tissue cultures performed.

Overall, results for seven persons met the definition of a confirmed case, 11 met the definition for probable cases, and one, having only cough and headache, did not meet either definition. Among the 18 attendees with probable or confirmed histoplasmosis, 16 sought care, including seven who were hospitalized and seven who were treated with itraconazole. The other two had self-limited disease. All recovered.

No statistically significant associations were found between hospitalization and either demographic characteristics or activities at the site. Younger attendees were more likely to be hospitalized, but this association was not statistically significant (chi-square test, p=0.084). Quantitative anti-*Histoplasma* antibody titers were not associated with either activities at the site or demographic characteristics.

## Actions Taken

All attendees were provided information on histoplasmosis and were encouraged to contact their physician if ill. Primary care providers statewide and Arkansas county health officers were notified of the outbreak and were reminded to consider histoplasmosis in the differential diagnosis of patients presenting with compatible symptoms. All children were evaluated by a pediatric infectious diseases specialist, either in person or via telephone.

What is already known on this topic?

The association of *Histoplasma capsulatum* with bird feces, bat guano, or disturbed ground has long been established.

What is added by this report?

This report describes efficient transmission of *H. capsulatum* in the setting of a bonfire of bamboo that was previously used as a blackbird roost. This bonfire was the only common exposure reported by all ill attendees.

What are the implications for public health practice?

Clinicians should be aware that exposure to a bonfire of bamboo in which birds have previously roosted might be a risk factor to consider when questioning patients with signs and symptoms of histoplasmosis.

In an effort to prevent recurrence of histoplasmosis, the owner had the home’s heating and air mechanical systems professionally cleaned and also planned to cut down the bamboo grove to lessen roosting. On request, he was provided current CDC recommendations for the use of formaldehyde in environmental decontamination with histoplasmosis (1). However, decontamination was not recommended by ADH because it was judged in this instance to be impractical and marginally effective.

### Editorial Note

Histoplasmosis is endemic in Arkansas and many states along the Ohio, Mississippi, and Missouri river valleys (2). This is the first outbreak of histoplasmosis associated with a bamboo bonfire reported in Arkansas. A previous report from Louisiana in 1980 linked histoplasmosis to the clearing of a field of bamboo measuring 40 feet by 70 feet that was heavily laden with blackbird feces (3). In that case, the trees were bulldozed to the middle of the field and burned, and all six workers became ill. Because all attendees in both outbreaks reported illness, this raises the possibility that heating of *Histoplasma* spores in conjunction with fire-related air currents might create an ideal mode of transmission. Additional research on heating and desiccation on mold particle size and infectivity might be warranted.

The findings in this report are subject to at least two limitations. First, persons who were ill with histoplasmosis-compatible symptoms but did not have definitive testing were included as meeting the definition for probable cases in the context of this outbreak, which might overestimate the case count. Second, because of the small number of cases and lack of variability in exposures reported, data from the investigation were insufficient to determine statistically significant findings relating an exposure at the site to acute histoplasmosis. The only exposure that was clearly associated with illness was attending the bonfire. However, because all attendees participated in the bonfire, the magnitude of association could not be estimated.

## Figures and Tables

**FIGURE:**
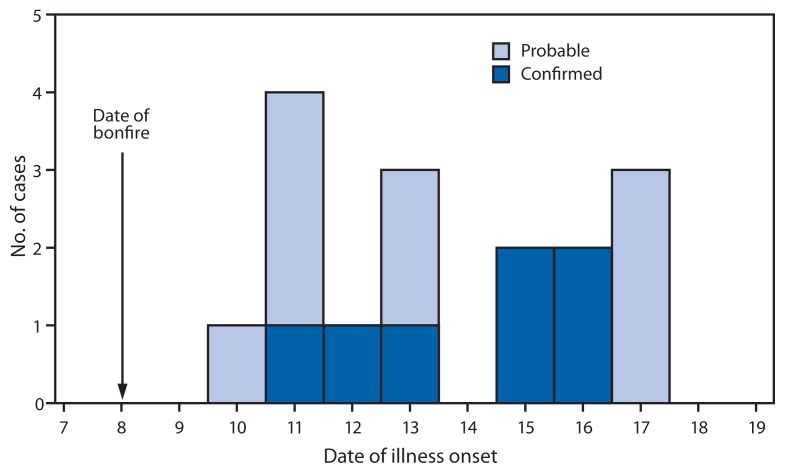
Illness onset among 16 persons^*^ with probable or confirmed histoplasmosis associated with a bamboo bonfire — Arkansas, October 2011 ^*^ Among 18 cases; two symptomatic attendees could not recall when their symptoms started.

**TABLE:** Characteristics of 18 persons who attended a bamboo bonfire and had signs and symptoms of suspected histoplasmosis — Arkansas, October 2011

Characteristic	No.	(%)
**Sex**		
Male	11	(61)
Female	7	(39)
**Age group (yrs)**		
<5	2	(11)
5–9	5	(28)
10–19	5	(28)
≥20	6	(33)
**Signs and symptoms**		
Fever (≥101°F [≥38.3°C])	15	(83)
Cough	12	(67)
Shortness of breath	11	(61)
Chest pain	9	(50)
Body aches	8	(44)
Vomiting	5	(28)

## References

[1] CDC (2004). Histoplasmosis—protecting workers at risk.

[2] Edwards LB, Acquaviva FA, Livesay VT, Cross FW, Palmer CE (1969). An atlas of sensitivity to tuberculin, PPD-B, and histoplasmin in the United States. Am Rev Respir Dis.

[3] Storch G, Burford JG, George RB, Kaufman L, Ajello L (1980). Acute histoplasmosis—description of an outbreak in northern Louisiana. Chest.

